# Advances in Genetic Diversity of Germplasm Resources, Origin and Evolution of Turnip Rape (*Brassica rapa* L.)

**DOI:** 10.3390/plants14152311

**Published:** 2025-07-26

**Authors:** Xiaoming Lu, Tianyu Zhang, Yuanqiang Ma, Chunyang Han, Wenxin Yang, Yuanyuan Pu, Li Ma, Junyan Wu, Gang Yang, Wangtian Wang, Tingting Fan, Lijun Liu, Wancang Sun

**Affiliations:** 1 College of Agronomy, Gansu Agricultural University, Lanzhou 730070, China; lxm955991@163.com (X.L.); 15352107840@163.com (T.Z.); 18894579003@163.com (Y.M.); hannn216@163.com (C.H.); 17393636612@163.com (W.Y.); puyy@gsau.edu.cn (Y.P.); wujuny@gsau.edu.cn (J.W.); yangg@gsau.edu.cn (G.Y.); fantt@gsau.edu.cn (T.F.); 2 State Key Laboratory of Aridland Crop Science, Gansu Agricultural University, Lanzhou 730070, China; mal@gsau.edu.cn; 3 College of Life Science and Technology, Gansu Agricultural University, Lanzhou 730070, China; wangw@gsau.edu.cn

**Keywords:** *Brassica rapa* L., morphological variation, origin and evolution, karyotype analysis

## Abstract

During a prolonged domestication and environmental selection, *Brassica rapa* has formed diverse morphological types during a cultivation process of up to 8000 years, such as root-type turnips (*Brassica rapa* var. *rapa*), leaf-type Chinese cabbage (*Brassica rapa* var. *pekinensis*), oil-type rapeseed (*Brassica rapa* L.), and other rich types. China is one of the origins of *Brassica rapa* L., which is spread all over the east, west, south, and north of China. Studying its origin and evolution holds significant importance for unraveling the cultivation history of Chinese oilseed crops, intraspecific evolutionary relationships, and the utilization value of genetic resources. This article summarizes the cultivation history, evolution, classification research progress, and germplasm resource diversity of *Brassica rapa* var. *oleifera* in China. Combining karyotype analysis, genomic information, and wild relatives of *Brassica rapa* var. *oleifera* discovered on the Qinghai–Tibet Plateau, it is proposed that *Brassica rapa* var. *oleifera* has the characteristic of polycentric origin, and Gansu Province in China is one of the earliest regions for its cultivation. *Brassica rapa* var. *oleifera*, originating from the Mediterranean region, was diffused to the East Asian continent through two independent transmission paths (one via the Turkish Plateau and the other via Central Asia and Siberia). Analyzing the genetic diversity characteristics and evolutionary trajectories of these two transmission paths lays a foundation for clarifying the origin and evolutionary process of *Brassica rapa* var. *oleifera* and accelerating the breeding of *Brassica rapa* var. *oleifera* in China. Despite existing research on the origin of *Brassica rapa* L., the domestication process of this species remains unresolved. Future studies will employ whole-genome resequencing to address this fundamental question.

## 1. Introduction

China is a major country in rapeseed cultivation, mainly growing three types: *Brassica rapa* L., *Brassica napus* L., and *Brassica juncea* L. Among them, *Brassica rapa* L. has the longest cultivation history. As an important oil crop in agriculture, the rapeseed oil produced by *Brassica rapa* L. is rich in various vitamins, and it is especially high in vitamin E, which is of great value to human health [1]. Different countries in the world have different names for *Brassica rapa* L. In Europe and America, *Brassica rapa* L. was called turnip rape [2], while *Brassica rapa* L. was called Yuntai or small rapeseed in China [3]. It mainly includes two varieties: *B. campestris* var. *oleifera* grows prostrate at the seedling stage with a swollen taproot; *B. chinensis* var. *oleifera* has a robust plant, well-developed stems and leaves, and thick and broad leaves [1]. *Brassica rapa* L. is a species of *Brassica rapa*, which belongs to the *Brassica rapa* family and includes Chinese cabbage and root turnip [4]. It is the most widely distributed and cultivated species in *Brassica rapa*. Due to the combined effects of geographical distribution, ecological environments, and selective pressures, *Brassica rapa* exhibits substantial diversity in morphology, genetic composition, and ecotypes. It also possesses a range of specialized traits, including cold tolerance, early maturation, and drought resistance, solidifying its status as a vital species within the Brassicaceae family. Li [5] and Liu [6] believed that China is the origin of *Brassica rapa* L. Initially, it was thought to be cultivated as a vegetable in North China; later, it was proposed that Shaanxi might be the origin of *Brassica campestris* var. *oleifera*. Moreover, through Random Amplified Polymorphic DNA (RAPD) molecular markers, it was indicated that the origin of *Brassica campestris* var. *oleifera* predated that of *Brassica chinensis* var. *oleifera*, the origin of winter turnip rape was earlier than that of spring rapeseed, and *Brassica chinensis* var. *oleifera* might have originated in regions such as Yunnan and Guizhou [3]. However, archaeological discoveries of rapeseed grains at the Dadiwan site in Gansu suggest that Gansu was the earliest region where rapeseed was cultivated. Tsunoda et al. [7] and Gómez et al. [8] argued that *Brassica rapa* L. originated in the Mediterranean, Northern Europe, or Western Europe and was introduced into China via Siberia and the Turkish Plateau [9]. At present, the origin of *Brassica rapa* L. remains inconclusive. The lack of clarity in understanding its origin not only hinders a complete analysis of the evolutionary context of this species but also underscores the urgency of systematic research. Investigating the origin, distribution, and domestication process of *Brassica rapa* holds significant implications for the conservation and utilization of its germplasm resources. However, the global distribution and complex evolutionary history of *Brassica* crops pose considerable challenges in deciphering their genetic architecture [10]. With the development of bioinformatics and gene sequencing technologies in recent years, whole-genome resequencing is regarded as a powerful tool that can address issues such as the population structure, origin, and domestication history, and adaptive evolution of species. As an important economic crop, the genomic research on *Brassica rapa* L. has also benefited from the advancement of this technology. This article synthesizes recent research findings on the origin, diversity, and utilization of *Brassica rapa* L., aiming to identify points of consensus and controversies, thereby laying the groundwork for further research into the origin and domestication of this species.

## 2. Results

### 2.1. The Centuries-Old Cultivation and Historical Trajectory of Brassica rapa in China

In the narrow sense, rapeseed includes three species: *Brassica rapa* L., *Brassica napus* L., and *Brassica juncea* L. Among them, *Brassica rapa* L., also known as Yuntai (2n = 20, AA), is one of the places of origin in China with a cultivation history of more than 8000 years [3,11]. It is also one of the five major oil and grain crops that originated in China. In the Western Han Dynasty tomb at Mawangdui, dating back approximately 5000 years, well-preserved mustard seeds were unearthed [12]. At the Banpo Neolithic Site in Shaanxi, which is about 7000 years old, a large number of carbonized mustard and cabbage seeds were excavated [13]. In 1979, at the Dadiwan Site in Gansu, dating to approximately 8000–12,000 years ago, grains of *Panicum miliaceum* from the Poaceae family and rapeseed seeds from the Brassicaceae family were discovered [14]. This represents the earliest evidence of rapeseed cultivation in China. Table 1 documents descriptions of rapeseed in various Chinese historical texts, demonstrating its extensive cultivation history in China. In 3000 BC, Xia Xiaozheng, the almanac of the Xia Dynasty, recorded that Yun was picked in the first month and Rong Yun in February, and Yun was the rape cultivated by later generations [15]. Historical documents, including Lv’s Spring and Autumn Annals, The Book of Poetry [16], Qimin Yaoshu, the Compendium of Materia Medica, Tiangong Kaiwu, and the Compendium of Materia Medicais, all document rapeseed, which was initially consumed as a vegetable. During the Han Dynasties (206 BCE–220 CE), rapeseed oil was widely used for culinary purposes. In the Popular Prose of the Eastern Han Dynasty, it was mentioned that rapeseed was planted in modern Gansu, Qinghai, Xinjiang, and Inner Mongolia. In the 13th century, farmers began cultivating rapeseed in winter-fallow fields, establishing a rapeseed–rice crop rotation system. In the 14th century, rapeseed cultivation expanded across southern China. The adoption of rapeseed seedling transplanting techniques resolved seasonal constraints in the rapeseed–rice rotation cycle in the 15th century. *Brassica rapa* L., a Brassicaceae oil crop, emerged as a major oilseed crop in China in the 17th century [15]. Although in the 1960s, *Brassica napus* L. was introduced into China, *Brassica rapa* L. and *Brassica juncea* L. remained the dominant cultivated varieties [17]. At the end of the 1960s, *Brassica rapa* L. was gradually replaced by *Brassica napus* L. because of the occurrence of virus disease and the failure to adopt disease-resistant breeding in time to solve the disease-resistant problem [18,19]. *Brassica napus* L. was introduced to China in the late 1930s, which had the advantages of virus resistance and high yield, and gradually became the main planting group in China in the 1970s [20]. Today, the cultivation of *Brassica rapa* L. is largely confined to Canada and Northern Europe, with minimal presence in Central Europe [21].

Research on *Brassica rapa* L. in China had already been documented in the early 1950s. Breeders conducted extensive efforts to collect, organize, and select from the abundant local variety resources of *Brassica rapa*. This work led to the development of superior cultivars such as ‘Wuyou1’ [22], ‘Xishui bai’ [18], ‘Wuhu104’ [23], ‘Baiyou1’ [24], and collaborative varieties like ‘Xiezuo1’ and ‘Xiezuo2’ [25], all of which played a positive role in improving yields. Since the late 1950s, under the one-sided pursuit of high yields, *Brassica rapa* L. was dismissed as having poor disease resistance, low productivity, and no longer being valuable for continued application [23]. From then on, the breeding of new *Brassica rapa* L. varieties was largely abandoned [23]. However, this traditional oilseed crop retains unique advantages over *Brassica napus* varieties in cold tolerance, delayed sowing adaptability, early maturity, poor soil tolerance, and drought resistance. Particularly under low soil fertility and drought conditions, *Brassica rapa* L. demonstrates significantly higher and more stable yields compared to its *Brassica napus* L. Therefore, although *Brassica rapa* L. has long been neglected, it remains agriculturally utilized to this day. Notably, its cultivation persists in high-altitude regions and even within major rapeseed production zones such as Sichuan and Anhui provinces. Furthermore, new cultivars continue to be developed and documented in academic literature.

In Shanxi and Qinghai provinces, researchers have successively developed *Brassica rapa* L. cultivars, including ‘Jinyou2’ [26], ‘Qingyou11’, and ‘Qingyou15’ [27]. In September 1992, the Agricultural Science Institute of Chizhou Prefecture, Anhui Province, bred the semi-winter type ‘Wanyou7’ from the Jiashan Small-seed Rapeseed germplasm [28]. Meanwhile, the Gansu Academy of Agricultural Sciences has cultivated the *Brassica rapa* cultivars ‘Longyou3’ and ‘Longyou4’ through systematic breeding programs [29,30]. The 21st century has witnessed groundbreaking advancements in *Brassica rapa* breeding. Gansu Agricultural University successfully developed the ‘Longyou7’ series—strong and tolerant winter rapeseed cultivars [31]—which have been widely adopted in northern China. Remarkably, these cultivars demonstrate winter survival capability even in Heihe, Heilongjiang Province, successfully addressing the historical challenge of winter rapeseed overwintering in northern regions. This breakthrough has driven rapid expansion of winter rapeseed cultivation across northwestern and northern China, markedly expanding the cultivation zone from the north of 35° N around Tianshui to 48° N Xinjiang and other places. In recent years, amid growing strategic imperatives to expand cultivation areas and enhance productivity of oilseed crops, coupled with ecological conservation requirements, *Brassica rapa* L.—particularly its cold, drought, and aline-alkali-tolerant traits—has undergone renewed scientific recognition. This traditional crop has consequently experienced revitalized agricultural value, demonstrating resurgent vitality in contemporary farming systems characterized by climate challenges and environmental constraints.

### 2.2. Genetic Diversity in Brassica rapa Germplasm Resources

*Brassica rapa* crops have accumulated substantial germplasm diversity through prolonged domestication and cultivation, encompassing essential vegetable morphotypes such as Chinese cabbage (*Brassica rapa* var. *pekinensis*), bok choy (*Brassica rapa* var. *chinensis*), turnip (*Brassica rapa* var. *rapa*), and caitai (*Brassica rapa* var. *parachinensis*), as well as oilseed crop variants exemplified by the *Brassica rapa* L. [32]. The collection, identification, and conservation of rapeseed germplasm resources conducted around 1996 gathered over 5200 accessions, of which 45.8% were *Brassica rapa* L. By the end of 2019, China had collected a total of 9681 germplasm resources, including 2847 accessions of *Brassica rapa* and 831 wild relatives [33]. These invaluable resources constitute a vital gene bank for research on the genetic diversity and breeding innovation of *Brassica rapa* crops. Their long-term cryopreservation is crucial for safeguarding against varietal degeneration, maintaining genetic diversity, and advancing the development of new cultivars. Furthermore, the application of molecular marker-assisted selection (MAS) technology enables efficient identification and elimination of hybrid plants, significantly reducing field workload and accelerating the breeding cycle. The main production areas of *Brassica rapa* L. are in China and the Indian subcontinent. In China, *Brassica rapa* comprises two distinct types: the Northern rapeseed and the Southern Chinese cabbage. Representative varieties of Northern rapeseed include ‘Xiaoriqi rapeseed’, ‘Menyuan small rapeseed’, and ‘Tianzhu small rapeseed’. Southern Chinese cabbage varieties primarily feature ‘Xinghua rapeseed’, ‘Dongkou sweet rapeseed’, and ‘Qixingjian rapeseed’ [34]. *Brassica rapa* L. in China exhibits abundant genetic diversity due to its extensive cultivation history and the significant ecological variations in its planting areas. Indian *Brassica rapa* L. includes three subspecies: yellow sarson (*Brassica rapa* subsp. *trilocularis*), brown sarson (*Brassica rapa* subsp. *dichotoma*), and toria (*Brassica rapa* subsp. *dichotoma*), which are distributed across the Indian subcontinent, including countries like India and Pakistan [35]. These types may have originated from either a wild population native to India or introduced varieties of turnip rape [36].

Genetic diversity in temperature sensitivity and growth period. Research has found that *Brassica rapa* L. in China includes ecotypes such as a strongly winter-type, winter, semi-winter, and spring types. Among these, strongly winter-type varieties require 80–90 days of vernalization treatment at 0–4 °C to complete the process, with a growth period reaching approximately 300 days [37,38]. In contrast, some spring germplasms such as ‘Tianzhu small rapeseed’ and ‘Menyuan small rapeseed’ can achieve vernalization even at temperatures above 20 °C without specific low-temperature treatment, exhibiting a significantly shorter growth period of only about 70 days [39]. Strongly winter-type varieties are cultivated as autumn-sown crops in northern regions, whereas short-growth-period germplasms such as ‘Tianzhu small rapeseed’ and ‘Menyuan small rapeseed’ are grown as summer rapeseed in the same areas following the harvest of wheat and legumes. Winter rapeseed exhibits the longest growth period, whereas spring rapeseed demonstrates the shortest [40]. Hu et al. [41] conducted growth period evaluation using 314 Tibetan *Brassica rapa* accessions, revealing the following maturity distribution: ultra-early-maturing varieties (growth period < 85 days) accounted for 9.55%, early maturing (85–115 days) 69.47%, medium maturing (115–145 days) 15.92%, medium–late maturing (145–160 days) 5.41%, and ultra-late maturing (>160 days) 0.64%. Chen et al. [42] conducted agronomic characterization of 179 Guizhou *Brassica rapa* local landrace accessions, revealing a total growth period ranging from 189 to 200 days, with accessions from high-altitude regions exhibiting prolonged growth periods compared to those from low-altitude counterparts. Yang et al. [43] performed comparative growth period analysis based on 1071 germplasm accessions encompassing *Brassica juncea*, *Brassica rapa*, and *Brassica napus*, revealing that *Brassica rapa* exhibited significantly shorter growth periods compared to both *Brassica juncea* and *Brassica napus*.

Excellent stress resistance. *Brassica rapa* L. exhibits outstanding stress resistance. The strong winter-type varieties cultivated by Gansu Agricultural University demonstrate exceptional cold resistance, enabling them to overwinter under extreme low temperatures of −30 °C. These varieties exhibit unique cold-resistant traits such as prostrate growth, depressed apical meristems, and early leaf senescence period [44]. Meanwhile, *Brassica rapa* L. demonstrates remarkable drought and saline-alkali resistance. Researchers from Gansu Agricultural University have screened superior saline-alkali-tolerant germplasms such as ‘SCKY-6-27’, which exhibits good growth in 0.6% saline-alkali soil conditions [45].

Superior adaptability. *Brassica rapa* exhibits an exceptionally broad distribution range, spanning severe cold regions (Tashan, Urumqi, Heilongjiang [46,47,48]) and warmer areas like Fujian. Its cultivation extends from Zengmu Reef at 3° N latitude to Mohe County at 53° N, and from the Pamir Plateau at 73° E longitude to the banks of the Ussuri River at 135° E. Notably, its adaptability surpasses all other rapeseed varieties and ranks among the most adaptable crops in China. *Brassica rapa* exhibits dual reproductive strategies, comprising both self-compatible (SC) and self-incompatible (SI) systems. Notable SC accessions include ‘Yellow sarson’ [49], ‘Huangyabai’ [50], ‘Qinghai Dahuang’ [51], ‘Tibetan yellow-seeded landraces’, ‘Guobu’, and ‘Pubacun’ [52]. Recent breakthroughs by Gansu Agricultural University revealed novel, strong winter-hardy SC lines [53]. Furthermore, multi-locular rapeseed accessions have been identified in Qinghai germplasms [54].

Diversity in botanical morphology, characteristic traits, and agronomic traits. As shown in Table 2, Strong winter and winter rapeseed exhibit prostrate seedling growth, accelerated subterranean development, enlarged hypocotyl diameter, and robust taproot systems [55]. Winter rapeseed displays purple heart leaves, dark or light green foliage, maintains prostrate growth before winter, and shows no bolting or budding, whereas spring rapeseed features green heart leaves, yellow-green foliage, erect seedling growth, and a strong spring growth habit [56]. Studies have shown that the biological characteristics of *Brassica rapa* L. have evolved from the semi-erect and erect seedling growth of wild types to the semi-erect, prostrate, and erect growth of cultivated varieties. Plant height has evolved from short to tall, and the color of the elongated stem has gradually evolved from purple to green. The main inflorescence length and single-plant yield of wild types are lower than those of cultivated varieties. The traits of basal leaves, stem leaves, and floral organs in wild and cultivated *Brassica rapa* have undergone both synchronous and asynchronous evolution [57].

Silique seed number, total siliques per plant, and thousand-seed weight constitute the primary yield-determining traits [58]. As evidenced by Table 2, the number of siliques per plant in winter-type and semi-winter-type *Brassica rapa* L. is similar and higher than that in spring-type *Brassica rapa* L. [40]. The thousand-seed weight of *Brassica rapa* L. typically ranges from 2.4 to 3.0 g, with exceptional specimens reaching up to 8.0 g [59]. Winter-type and semi-winter-type *Brassica rapa* accessions exhibited lower thousand-seed weights compared to spring-type varieties [40]. While winter-type and semi-winter-type rapeseed showed greater plant height than spring-type counterparts, spring-type and semi-winter-type accessions produced fewer primary branches than winter-type *Brassica rapa*. Winter-type and semi-winter-type varieties exhibit a higher silique number per plant than spring-type rapeseed [40].

Diversity in quality traits. There are excellent materials with high oil content, high protein content in cake meal, and low cellulose content in China [60,61]. The oil content is related to the seed coat color. Rapeseed has yellow-seed coats [62], such as ‘Jianghuangzhong’ and ‘Dahuang’, and the oil content and protein content of yellow-seed lines are higher than those of black-brown-seed rapeseed [2]. The oil content of spring-type *Brassica rapa* L. in Tibet is higher than that in other regions of China. Cultivars exceeding 50% oil content include ‘Longzi’ (51.60%) from Lhünzê County, ‘Pubar’ (51.22%) from Nyêmo County, and ‘Tangluohong’ (50.32%) from Maizhokunggar County [63]. Landraces of *Brassica rapa* from the Qinghai-Tibet and Yunnan-Guizhou Plateaus exhibit seed oil content reaching up to approximately 50% [62]. The protein content of rapeseed germplasm resources in China ranges from 13% to 36%. The protein content of *Brassica rapa* L. is 24.59%, similar to that of *Brassica napus* L. (24.70%), but lower than that of *Brassica juncea* L. (27.45%) [64]. Glucosinolate profiles also exhibit substantial variability. To date, over 200 distinct glucosinolate types have been identified within the Brassicaceae family, with approximately 20 predominant forms commonly detected in *Brassica* oilseed crops [65]. Glucosinolate compounds are mainly composed of three parts: glycoside, sulfonic acid oxime, and side chain groups. According to the differences in side chain groups, they can be further divided into aliphatic, indolic, and aromatic types [66]. Glucosinolates are present in the roots, stems, leaves, and seeds of rapeseed, with the highest content found in the seeds [67]. The content of glucosinolates is influenced by factors such as variety, growth environment, and distribution position. Kim et al. [68] found that exogenous sulfur application increased the glucosinolate content in leaves of *Brassica rapa* L., indicating that the synthesis of glucosinolates can be promoted by applying sulfur from external sources. Ma et al. [69] identified nine glucosinolate components in 323 *Brassica rapa* accessions using high-performance liquid chromatography (HPLC), with gluconapin as the predominant constituent. Notably, unique accessions such as ‘2023xzbc-146’ exhibited elevated proportions of =progoitrin. When enzymatically degraded, this component yields products known to cause goiter in animals while simultaneously exhibiting molluscicidal activity, suggesting its potential as a botanical molluscicide source [70]. The indolic glucosinolate content in strong winter-type (11.50 µmol·g^−1^) materials is higher than that in winter-type (7.60 µmol·g^−1^), semi-winter-type (6.77 µmol·g^−1^), and spring-type (3.87 µmol·g^−1^) materials. Additionally, materials from the Yangtze River Basin exhibit higher indolic glucosinolate content than those from regions such as the Huanghuai area, Tibet, and Gansu [69].

### 2.3. Diversity in the Utilization of Brassica rapa

Important oil crops and protein crops. Rapeseed oil ranks as the world’s third most produced vegetable oil, following soybean and palm oils. Characterized by a high proportion of unsaturated fatty acids with balanced n-3 and n-6 ratios, it contains abundant bioactive compounds, including phenolic acids, phytosterols, and carotenoids, establishing *Brassica* species as economically vital oil crops [71]. Moreover, rapeseed serves dual purposes as a source of edible oil for human consumption and protein-rich animal feed [72].

Pioneer crop in planting system reform. *Brassica rapa* L. is ideally suited for the one-year-two-cropping or two-year-three-cropping farming systems due to its flexible growth period [73]. Pan [39] demonstrated that early-maturing cultivars like ‘Tianzhu’ and ‘Menyuan rapeseed’ complete their life cycles in approximately 70 days. They can be relay-planted after spring or autumn crops to maximize land use in double-cropping regions. Conversely, strong winter-type *Brassica rapa* cultivars in northern China mature between late May and mid-June, leaving sufficient thermal units for subsequent cultivation of summer maize, soybean, peanut (*Arachis hypogaea* L.), proso millet (*Panicum miliaceum* L.), and foxtail millet (*Setaria italic* L.). This adaptability renders *Brassica rapa* indispensable in double-cropping regions and high-altitude agroecosystems.

Plant mulch and eco-environmental crop. Northern winter-type *Brassica rapa* L. serves as an excellent winter-spring cover crop, ideally suited for autumn intercropping and ground coverage in orchards of the Loess Plateau. It effectively retains soil moisture, suppresses dust, and acts as a vital plant mulch and eco-environmental crop [74,75,76]. As a winter green manure cover crop, it improves soil structure, increases organic matter content, and significantly enhances yields of subsequent crops. Studies on returning green manure crops such as winter-type *Brassica napus* and *Brassica rapa*, *Vicia villosa*, and *Orychophragmus violaceus* to the field have shown that turning under winter-type *Brassica rapa* significantly boosts the yield of subsequent maize, outperforming other green manure crops [77]. Additionally, *Brassica rapa* exhibits higher early-stage biomass than *Brassica napus*, enhancing its green manure value [20]. Zhang et al. [78] conducted a study where the winter-type *Brassica rapa* variety ‘Tianyou8’ was relay-planted after spring wheat harvest. After turning under the green manure, the biomass and grain yield of spring wheat increased by 23.03% and 17.68%, respectively.

Vegetable, medicinal, nectar, and ornamental plant. *Brassica rapa* L. has multiple functions, including oil production, vegetable consumption, floral utilization, nectar production, and green manure. Notably, the oil–vegetable dual-purpose ecotypes of *Brassica rapa* represent a unique agricultural heritage in China. The Lintao rapeseed in Gansu can be used both as a vegetable and for oil extraction [79], representing an evolutionary stage of *Brassica rapa* L. in China. The seeds of *Brassica rapa* exhibit high glucosinolate content, demonstrating pest resistance, antibacterial properties, and antitumor effects [67], thereby serving as an organic botanical pesticide. Additionally, its early flowering nature makes it the first bloom for honeybees, positioning it as a vital nectar source plant and landscape species.

Industrial raw materials. Rapeseed oil from *Brassica rapa* L. holds significant industrial value and is widely used in industries such as paint, plastics, cosmetics, and pharmaceuticals [80], as well as in biodiesel production [81]. Currently, the Qinghai-Tibet Plateau region has the potential to develop biodiesel production, which could drive the growth of the rapeseed industry through biodiesel-related businesses [81].

### 2.4. Advances in the Origin Research of Brassica rapa

#### 2.4.1. Phylogenetic Relationships Among *Brassica rapa* Crops

The *Brassica rapa* complex exhibits remarkable morphological diversity across its cultivated forms, including heading Chinese cabbage (*Brassica rapa* subsp. *pekinensis*), pak choi (*Brassica rapa* subsp. *chinensis*), turnip (*Brassica rapa* subsp. *rapa*), and yellow sarson. This extensive phenotypic plasticity significantly challenges the identification of evolutionary origins and diversity centers through morphological analysis alone [82,83]. Archaeological evidence indicates that the rapeseed varieties cultivated in ancient China gradually evolved from vegetable crops. *B. campestris* var. *oleifera* originated from the ancient cultivated plant yuntai, while *B. chinensis* var. *oleifera* developed from Chinese cabbage [84]. In this regard, Tong [15] proposed that *Brassica rapa* L. originated from the Chinese cabbage cultivated in the northwestern region of China, and another type of *Brassica rapa* evolved from pak choi, which is called *B. chinensis* L. Through comprehensive analysis integrating morphological, isoenzyme, seed protein, and RFLP (Restriction Fragment Length Polymorphism) marker results, *Brassica rapa* is classified into root-type turnips in Europe, Central Asia, and India, oil-use types in India, and leaf vegetable types in East Asia. Wild *B. rapa* originating in Europe served as the common ancestor of both *Brassica rapa* crops and *Brassica oleracea*, with wild *Brassica rapa* being domesticated successively to form two primitive types: turnip and turnip rapeseed [85]. Molecular evidence shows that Chinese turnip, European turnip, and sarson are early differentiations from the common ancestor of *Brassica rapa*, suggesting that tuber crops originated earlier than oil crops [83]. Tanhuanpää et al. [35] divided the population into three clusters based on 209 SNP markers from 61 accessions of *Brassica rapa*, and their grouping corresponded to morphological types and flowering habits, indicating that morphologically similar crops are more closely related. Zhao et al. [86] used AFLP markers to analyze the genetic diversity of *Brassica rapa* crops. The results indicated that different subgroups correspond to distinct morphotypes. Within the Middle Eastern and European *Brassica rapa* crops, accessions of different types from the same region exhibited closer genetic relationships than accessions of the same type from different regions. This suggests that these two regions either represent independent centers of origin or experienced independent domestication events. It is worth noting that Li [87] studied 30 *Brassica rapa* vegetables and found that the Indian-originated yellow sarson, a type of rapeseed, was significantly differentiated from other types of *Brassica rapa* vegetables. Pak choi, Chinese cabbage, European turnip, and Wutacai formed a close evolutionary clade. This result not only confirms that yellow sarson is a unique oil-use type of *Brassica rapa* crops independently domesticated in the Indian subcontinent but also implies that South Asia may serve as a secondary diversity center for this species, providing key evidence for the hypothesis of multiple origins of *Brassica rapa* L.

#### 2.4.2. Geographic Origins

**Figure 1:**
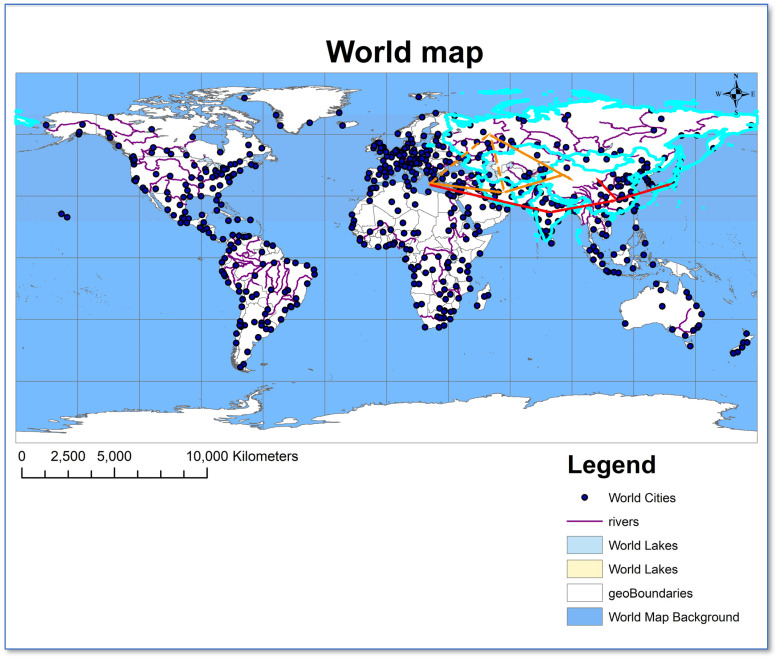
Hypothetical origin and dispersal routes of oil-type rapeseed (*Brassica rapa* L.). Note: Dashed lines indicate hypothetical relationships; solid lines represent empirically supported evolutionary pathways. The light blue border represents the countries traversed along the route.

Based on current research, rapeseed has two major centers of origin. The first is the Asian center, primarily encompassing China and India [88], which served as the origin center for Chinese Cabbage and *Brassica rapa* L. [6]. Carbonized seed remains discovered at the Dadiwan site in Tianshui, Gansu Province (8000 years ago) and the Banpo site in Xi’an, Shaanxi Province (6800 years ago) indicate that Gansu and Shaanxi were the earliest regions for *Brassica rapa* L. cultivation. The second is the European center, which is recognized as the origin center for *Brassica oleracea*, *Brassica rapa*, black mustard (*Brassica nigra*), and *Brassica napus* L. [88]. Due to differences in geographical distribution and ecological environments, *Brassica rapa* in northern and southern China exhibit variations in morphological characteristics and reproductive cycles. *Brassica rapa* L. in China is divided into two major types: *B. campestris* var. *oleifera* and *B. chinensis* var. *oleifera*. It is believed that *B. campestris* var. *oleifera* originated earlier than *B. chinensis* var. *oleifera*, with Shaanxi and Gansu possibly being the origin places of *B. campestris* var. *oleifera* [6]. *B. chinensis* var. *oleifera* may have originated in regions such as Yunnan, Guizhou, and Sichuan [3,89]. Liu [6] proposed that rapeseed has a polyphyletic origin, and China is one of the origin centers for both *Brassica rapa* L. and *Brassica juncea* L., with *Brassica rapa* L. originating in the Central and Northwest regions of China. Wang et al. [90,91] collected wild *Brassica rapa* germplasm resources on the Tibetan Plateau and proposed that the Tibetan Plateau is one of the origin centers of rapeseed worldwide. The cultivated species of *Brassica rapa* are divided into two subspecies: one group comprising turnips and oil-use types, primarily distributed in Europe, Central Asia, and India; the other group consisting of leafy vegetable types, mainly found in East Asia. This suggests a distant genetic relationship between leafy vegetables and turnips [92]. Some studies indicate that *Brassica rapa* originated in the Mediterranean Basin, Northern Europe, or Western Europe [7,8]. The turnip, the earliest variety evolved from wild ancestors [9], spread via the Anatolian Plateau to Central Asian regions such as Iran and Afghanistan, and was subsequently introduced to the Chinese mainland from Central Asia and Siberia around the beginning of the Common Era (CE). Song et al. [36] proposed that *Brassica rapa* originated in Europe, dispersed through the Middle East to India and southern China, forming distinct wild populations, and subsequently spread northward from southern regions. Vaughan [93], Song et al. [85], Chen et al. [94], Guo et al. [95], and He et al. [3] demonstrated through RFLP, Random Amplified Polymorphic DNA (RAPD), and Amplified Fragment Length Polymorphism (AFLP) molecular markers that *Brassica rapa* has two independent origin centers in Europe and Asia. Guo et al. [82] investigated the origins and genetic diversity centers of *Brassica rapa* using 51 pairs of SSR primers and 715 polymorphic loci. The results demonstrate that *Brassica rapa* originated in the Old World, including Southwest Asia, the Mediterranean Basin, and temperate Europe, with genetic diversity centers located in East Asia, along ancient Asian trade routes, and in North and South America. However, a key unresolved consensus is whether the delineation of genetic and population structures reflects geographic origin or morphological origin [10]. Building on prior research, the origin hypothesis and evolutionary pathway of *Brassica rapa* are illustrated in Figure 1. This species originated primarily in the Mediterranean Basin as its main origin center and dispersed to the East Asian continent through two independent migration routes. However, the specific geographic corridors and genetic evolutionary mechanisms underlying the two potential migration routes—one from the Mediterranean to Siberia, and the other from the Iranian Plateau to Siberia—still require systematic elucidation through comparative genomics and archaeobotanical studies.

#### 2.4.3. Karyotype-Based Analysis

The karyotype refers to the phenotypic characteristics of a complete chromosome set during the metaphase of mitosis. Through karyotype analysis, evolutionary trends of species can be investigated. Generally speaking, the karyotypic evolutionary trajectory across the plant kingdom progresses from symmetrical to asymmetrical configurations [96,97]. As shown in Table 3, Stebbins [98] classified karyotypes across the biological realm into 12 distinct types (Type A representing symmetrical configurations, Type B intermediate configurations, and Type C asymmetrical configurations) based on chromosome arm ratios and length ratios, with parameter ranges further divided into four segments. In 1930, Levitzky [99] first proposed the concepts of karyotypic symmetry and asymmetry, and noted that the degree of karyotypic asymmetry in flowering plants could serve as an indicator of evolutionary advancement in karyotypes. Li et al. [100] conducted karyotype analyses on eight accessions of Tibetan, yellow-seeded *Brassica rapa*, incorporating karyotype asymmetry indices. The results suggested that Tibetan, yellow-seeded *Brassica rapa* occupies a relatively primitive position in species evolution with a slower evolutionary rate. Cytological comparisons between the brown-seeded cultivar ‘Garang’ and the yellow-seeded cultivar ‘Xueba Yellow Rapeseed’ revealed that the brown-seeded cultivar exhibits a higher evolutionary status, classified as intermediate Type 2B, while the yellow-seeded ‘Xueba Yellow Rapeseed’ demonstrates lower evolutionary advancement, retaining a more primitive symmetrical Type 1A configuration. Cheng [57] performed karyotype analyses on Tibetan cultivated *Brassica rapa* and wild *Brassica rapa*. The results indicated that the wild *Brassica rapa* from Tibet possesses eight metacentric chromosomes and two submetacentric chromosomes, exhibiting a Type IIA karyotype characterized by higher symmetry and evolutionary primitiveness. In contrast, the cultivated *Brassica rapa* contains five metacentric and five submetacentric chromosomes, with a Type IIB karyotype classified as a more asymmetrical type. These karyotypic analyses elucidate the evolutionary stages of the germplasm materials.

#### 2.4.4. Analysis Based on Genomic Information

##### High-Throughput Sequencing of *Brassica rapa*

The origin and evolution of a plant group describe its formation and developmental processes. These are studied through comparative morphology, fossil evidence, and molecular biology methods. Notably, the advent of molecular biology techniques has significantly enhanced the reliability of origin and evolutionary analyses [101]. With the rapid advancement of sequencing technologies and the reduction in costs, high-throughput sequencing (HTS) has been achieved for multiple crop species, along with the assembly of reference genomes. These advancements serve as invaluable resources for genetics and genomics research [102]. As shown in Table 4, the doubled haploid *Brassica rapa* ‘Chiifu-401-42’ was the first reference genome to undergo whole-genome sequencing in the *Brassica* [103]. Subsequently, whole-genome sequencing was performed on yellow sarson [104,105], followed by the assembly of the genome of the winter-type *Brassica rapa* cultivar ‘Longyou7’ [102]. This serves as a high-quality reference genome for winter-type *Brassica rapa*, facilitating the discovery and functional validation of cold-tolerance candidate genes in this species. The advancement of HTStechnologies has provided opportunities to elucidate the phylogenetic relationships and domestication processes among *Brassica* crops [106]. Qi et al. [106] analyzed high-throughput data from 126 global *Brassica rapa* accessions, classifying the species into five major groups and supporting a European-Central Asian origin for *Brassica rapa*. The South Asian and East Asian groups—including pak choi, Chinese cabbage, and yellow sarson—were identified as likely monophyletic lineages, while rapeseed and brown sarson may have polyphyletic origins. In a separate study, Cheng et al. [83] performed resequencing of 199 *Brassica rapa* and 119 *Brassica oleracea* accessions, revealing that sarson and turnip occupy basal positions in the phylogenetic tree, whereas Chinese cabbage is positioned at the most distal branch. This suggests that sarson and turnip represent early diverging lineages ancestral to modern *Brassica rapa* crops.

#### 2.4.5. Advances in Cytoplasmic Genome Research

##### Progress in Mitochondrial Genome Evolution Studies

The mitochondrial genome is commonly used for phylogenetic analysis of species due to its high conservation [107]. Recent studies have revealed that frequent genetic exchange among nuclear, mitochondrial, and chloroplast genomes is a common phenomenon during species evolution [108]. To date, mitochondrial genome assemblies within the *Brassica* genus have been completed for the following species: *Brassica rapa* var. *purpuraria* [108], *Brassica napus* L. [109,110], *Brassica rapa* [111], yellow sarson [87], *Brassica juncea* L. [111,112], *Brassica oleracea* [111], *Brassica carinata* [111], Kohlrabi (*Brassica oleracea* var. *gongylodes* L.) [113], *Brassica nigra* [114]. Chang et al. [111] revealed through comparative mitochondrial genome analysis and evolutionary studies that the cytoplasmic origins of *Brassica juncea* and *Brassica napus* are primarily derived from *Brassica rapa*. Heng [115] compared the mitochondrial genomes of *Brassica rapa* (cam cytoplasm), *Brassica juncea*, and *Brassica napus* (pol CMS cytoplasm), revealing strong collinearity among them, which indicates high similarity in their mitochondrial genomes. Cluster analysis further grouped the cytoplasmic genomes of *Brassica rapa* (cam), *Brassica juncea*, *Brassica napus* (pol CMS and nap cytoplasms), and *Brassica oleracea* (ole cytoplasm) into a single clade. This suggests a close genetic distance between the A and C genomes. With the advancement of mitochondrial genome sequencing, we are not only gaining deeper insights into the origin and evolution of mitochondria, but also uncovering and applying novel mitochondrial genotypes and cytoplasmic type-specific markers [115]. Liu [116] conducted a divergence time analysis based on mitochondrial genomes of six *Brassica* species. The study revealed that *Arabidopsis* and *Brassica* diverged approximately 16 to 20 million years ago (Mya). Subsequently, *Brassica nigra* diverged from *Brassica rapa* and *Brassica oleracea* between 8.3 and 19 Mya. Finally, *Brassica rapa* and *B. oleracea* diverged around 0.5 to 7.4 Mya. These findings are consistent with evolutionary relationships inferred from nuclear genome analyses. To date, only the mitochondrial genome of yellow sarson has been assembled, which exhibits unique characteristics [87]. No similar mitochondrial genomes have been identified in other *Brassica* species. Future studies should focus on assembling additional mitochondrial genomes of *Brassica rapa* to determine whether multiple genome types exist and to assess potential differences among its variants.

##### Progress in Chloroplast Genome Evolution Studies

The chloroplast genome exhibits maternal inheritance and follows a relatively independent evolutionary pathway, enabling the construction of phylogenetic trees and elucidation of plant evolutionary history without reliance on additional datasets [117]. Lü [117] conducted specific amplification using 19 pairs of chloroplast genome-specific SSR primers on 53 accessions spanning 13 species across seven genera. The results indicated close phylogenetic relationships among *Brassica rapa*, *Brassica juncea*, and *Brassica napus*. Based on chloroplast DNA marker analyses, *Brassica* species were divided into two clades: one comprising *Brassica nigra*, and the other including *Brassica rapa* and *Brassica oleracea*. In another study, Gao [9] amplified 63 *Brassica rapa* accessions using 33 chloroplast simple sequence repeat (cpSSR) primers derived from the chloroplast genome. However, no distinct banding patterns were observed among the different *Brassicarapa* types, suggesting limited intra-species variation. Wu et al. [118] assembled the chloroplast genome of *Brassica rapa* L. and revealed through phylogenetic analysis that the accession ‘18R-1’ shares the closest evolutionary relationship with pak choi. Li [87] analyzed the chloroplast genome phylogeny and found that there are two different types of chloroplast genomes in *Brassica rapa*, namely the common Chinese cabbage type and the special Italian sprouting broccoli type. The study also revealed that *Brassica napus* L. originated from two independent hybridization events, with its maternal lineages derived from the yellow sarson and Italian sprouting broccoli. Notably, yellow sarson clustered together with the rapeseed chloroplast genome (KM454975.1) and showed good collinearity with most chloroplast genomes of *Brassica rapa* species. This result further supports the critical role of *Brassca rapa* as an important maternal donor in the hybrid evolution of *Brassica* crops.

## 3. Discussion

China boasts abundant germplasm resources of *Brassica rapa* L., which exhibit diverse uses and harbor significant unique genetic traits [119]. Winter turnip rape possesses excellent stress-resistant traits, such as being able to withstand extremely low temperatures and thrive in 0.6% saline-alkali soil. These superior stress-resistant traits give *Brassica rapa* significant value in production, making it possible to breed varieties that are cold-tolerant, saline-alkali-tolerant, drought-tolerant, and adapted to suitable growing seasons. Studying its origin and evolutionary processes is critical for the exploration and utilization of these specialized germplasm resources. Research indicates that there are three prevailing hypotheses regarding the origin of *Brassica rapa* L.: a Chinese origin, a European origin, and a Mediterranean Basin origin. Additionally, the unique geographical conditions of the Tibetan Plateau have driven the adaptive distribution of wild relatives, establishing it as one of the global centers of origin for rapeseed. Wild relatives of *Brassica rapa* distributed on the Tibetan Plateau exhibit the most diverse bolting stem and leaf growth habits, with wild *Brassica rapa* demonstrating the strongest cold tolerance [59]. Additionally, wild rapeseed has been discovered in Yunnan Province, China. This material is classified as winter-type *Brassica rapa* and has a growth period of 154–172 days [120]. Current research demonstrates that China possesses both wild types of *Brassica rapa* and primitive varieties such as turnips, Chinese cabbage, and turnip greens. The mitochondrial genome of Chinese *Brassica rapa* L. shows close affinity to the chloroplast genome of pak choi, suggesting that Chinese *Brassica rapa* may have evolved from pak choi. Meanwhile, Cheng et al. [83] revealed through resequencing 199 accessions of *Brassica* crops that sarsons and turnips occupy basal branching positions in the phylogenetic tree. This indicates that sarson and turnips represent early differentiations from the ancestral lineage of *Brassica* crops, implying the Indian subcontinent may be one of the potential centers of origin for *Brassica rapa*. Currently, only the mitochondrial genome of Yellow Sarson has been assembled [87]. Its mitochondrial genes exhibit distinct features. Research on the evolution of *Brassica rapa* based on mitochondrial genome information is relatively scarce. Subsequent studies could focus on this area to investigate the evolutionary process of *Brassica rapa*.

Based on current research findings, it remains unclear whether *Brassica rapa* has a monophyletic or polyphyletic origin. Key unresolved questions include whether the divergence between the northern and southern ecotypes of *Brassica rapa* in China is driven by geographical environmental factors or distinct species origins, as well as the temporal sequence of their divergence. To address these gaps, further investigation and more evidence are needed to support conclusions. This necessitates expanded collection of *Brassica rapa* germplasm resources and the application of forward genetics to resolve these evolutionary ambiguities. With the increasing emphasis on breeding work of *Brassica rapa* L. and the development of third-generation sequencing technologies and mining of multi-omics data, analyzing the evolutionary processes of traits such as yellow seeds, multi-locular pods, short growth period, cold resistance, and drought resistance in *Brassica rapa* will help clarify its origin and evolutionary history.

## 4. Conclusions

To sum up, *Brassica rapa* L. is an important oil crop, protein crop, eco-environmental crop, and industrial raw material, playing a significant role in agriculture and industry. It exhibits excellent stress resistance, diverse reproductive modes, diverse qualities, and diversity in botanical morphology and agronomic traits. Research findings from archaeology, transmission routes, genetics, genomics, and other fields have revealed that *Brassica rapa* L. has the characteristics of a multi-center origin. Therefore, studying its origin and evolutionary process is of great significance for clarifying the evolutionary relationships among different origin centers, analyzing its domestication mechanisms and adaptive evolution laws, and can also provide theoretical support for the innovative utilization of subsequent germplasm resources.

## Figures and Tables

**Table 1:** Historical investigation of rapeseed cultivation [13,15,16].

Title of Work	Era	Description of Rapeseed
Xia Xiaozheng [15]	3000 BCE (Xia Dynasty)	Gather rapeseed in the first lunar month, and it blooms in the second lunar month.
The Book of poetry [16]	11th–6th century BCE	Pluck the turnip and the radish, but do not judge them by their roots alone.
Lv’s Spring and Autumn Annals [15]	3rd century BCE (Warring States Period)	The finest vegetables include the rapeseed from Yanghua.
Popular Prose [15]	The late Eastern Han Dynasty (184 CE–220 CE)	Rapeseed is historically termed Barbarian Vegetable. Legend attributes its name to the Yuntai Rong tribe beyond the northern frontier, who were said to have first cultivated this plant.
Qimin Yaoshu [15]	6th century CE (Northern Wei Dynasty)	For planting mustard seeds, Sichuan mustard, and rapeseed, they should all be sown in the second or third month when there is favorable rainfall. In times of drought, plant them in ridged beds with irrigation. They will ripen and be ready for seed harvest in the fifth month.
Tiangong Kaiwu [15]	Ming Dynasty (1637 CE, by Song Yingxing)	Rapeseeds are placed into a cauldron and stir-fried over gentle heat until aromatic, then crushed and steamed.
The Compendium of Materia Medicais [13]	Song Dynasty (11th century CE, compiled by Su Song)	Rapeseed bears a slight resemblance to Chinese cabbage (*Brassica rapa* var. *pekinensis*) in shape, with green leaves featuring fine serrations.
Compendium of Materia Medica [15]	Ming Dynasty (1593 CE, by Li Shizhen)	In the bitterly cold lands of Qiang, Long, Di, and Hu, this vegetable is widely cultivated during the winter months for its ability to endure frost and snow.

**Table 2:** Comparative analysis of botanical traits between winter and spring *Brassica rapa.*

Traits	Winter-Type *Brassica rapa*	Spring-Type *Brassica rapa*
Botanical Traits	Prostrate growth at the seedling stage, relatively rapid growth of underground parts, large root collar diameter, and well-developed taproot; purple heart leaves, no bolting or budding before winter.	Green heart leaves, yellowish-green leaf color, Erect seedling growth habit and strong spring growth type.
Agronomic Traits	Exhibit similar and abundant total silique numbers per plant.	Exhibits significantly lower total silique numbers per plant.
	Exhibit lower thousand-seed weights.	Exhibits significantly higher thousand-seed weights.
	Exhibit greater plant height compared to spring ecotypes.	Exhibits significantly reduced plant height.
	Develops a greater number of primary branches.	Develops significantly fewer primary branches.

**Table 3:** Karyotype classification [97].

Largest Chromosome		Percentage of Chromosomes with Arm Ratio > 2:1			
	Smallest Chromosome	0.0	0.01–0.50	0.51–0.99	1.0
<2:1		1A	2A	3A	4A
2:1–4:1		1B	2B	3B	4B
>4:1		1C	2C	3C	4C

**Table 4:** Reference genomes of *Brassica rapa.*

Species	Cultivar	Release Year	Reference
*Brassica rapa*	Chiifu-401-42	2011	Wang et al., 2011 [103]
*Brassica rapa* Z1	Yellow sarson	2018	Belser et al., 2018 [105]
*Brassica rapa* L.	Longyou-7	2022	Wu et al., 2022 [102]

## Data Availability

Data from this study can be found in the article.
